# Taste Enhancement in Japanese Black Wagyu Beef Fed With Sake Lees: Insights From Metabolomic and Sensory Evaluations

**DOI:** 10.1002/fsn3.70839

**Published:** 2025-08-25

**Authors:** Hitomi Shikano, Kazuki Komatsu, Fumiya Koga, Meguru Hara, Kazuaki Yoshinaga, Naoto Ishikawa, Shu Taira

**Affiliations:** ^1^ Faculty of Food and Agricultural Sciences Fukushima University Fukushima Japan; ^2^ Livestock Research Centre, Fukushima Agricultural Technology Centre Fukushima Japan; ^3^ Fukushima Prefecture Kenpoku Livestock Hygiene Service Center Fukushima Japan

**Keywords:** livestock, meat, omics analysis

## Abstract

This study investigated the effects of sake lees (SL) supplementation on the taste characteristics of Japanese Black Wagyu beef. Analytical sensory testing and taste sensor analysis demonstrated significantly higher sweetness scores in the SL‐fed group. Liquid chromatography–mass spectrometry (LC–MS) identified increased levels of amino acids, nucleotides, and glucose 1‐phosphate, compounds associated with sweetness and umami perception. Metabolomic pathway analysis indicated upregulation of the tricarboxylic acid (TCA) cycle and purine metabolism in SL‐fed cattle, suggesting enhanced energy metabolism and glycogen accumulation. These metabolic changes were consistent with sensory data, highlighting the potential of SL as a functional feed additive for improving beef palatability. This integrative approach combining sensory and chemical profiling provides new insight into taste enhancement mechanisms in livestock products.

## Graphical abstract

Taste enhancement in Japanese black Wagyu (JBW) beef fed with Sake Lees: Feeding sake lees (SL) to JBW cattle altered beef metabolite profiles, as revealed by metabolomics and taste sensor analysis. Elevated glucose 1‐phosphate and amino acids were associated with enhanced sweetness, consistent with sensory evaluation. These findings highlight SL as a potential feed additive to improve beef palatability.

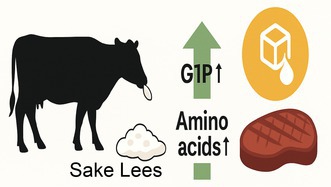

## 1 Introduction

Sake lees (SL), a traditional byproduct of Japanese sake production, have recently drawn attention as a promising functional feed ingredient due to their rich nutritional composition, including amino acids, vitamins, dietary fiber, and yeast‐derived compounds (Tsutsui et al. 1998; Mikami et al. 2020). Despite this potential, SL are still primarily used as cooking seasonings in Japan, and a significant portion remains unutilized or discarded (Yamato et al. 2020). Thus, novel applications that harness the biochemical benefits of SL are needed.

Previous studies have shown that SL supplementation can improve growth performance and meat traits in poultry and calves (Nishikawa et al. 2024; Ito et al. 2022), potentially due to its content of bioactive components such as amino acids, selenium, zinc, and prebiotics (Katsumata et al. 2023). However, research focusing on SL supplementation in beef cattle, particularly Japanese Black (Wagyu) known for its highly marbled meat (Ueda et al. 2007; Inoue et al. 2017), remains limited. Moreover, little is known about the biochemical mechanisms through which SL feeding might affect the taste profile of beef.

Beef carcasses are evaluated based on both estimated yield grade and meat quality grade, the latter of which is determined by several morphological factors, including the beef marbling score (BMS No.), meat color, and fat color. Among these, BMS No. is one of the most important classification criteria, as it reflects the degree of fat intermixture in the intramuscular fat. Grade A5, the highest grade in Japan, is classified as having a BMS No. of 8 or higher. In addition, rating systems have been introduced in various countries, all of which consider marbling and intramuscular fat content to be one of the main factors in evaluating the eating quality of beef as it relates to consumer satisfaction (Konarska et al. 2017).

Sensory analysis, which evaluates how taste is perceived when humans consume the product, is another critical method for assessing beef eating quality and can be classified into two types: analytical sensory testing and consumer sensory testing (Okumura et al. 2012; Suzuki et al. 2017). Analytical sensory testing involves trained panelists who objectively evaluate specific attributes, while consumer sensory testing relies on untrained consumers and often includes more subjective evaluations, making it useful for market research.

Physicochemical analysis is an established method for evaluating beef palatability and typically includes assessments of moisture content, protein content, fat content, centrifugal water‐holding capacity, heat loss, and shear force value (Qamberani 2024). Additionally, analyses of amino acid quantification and fatty acid composition are widely conducted. While these analytical approaches provide valuable insights into eating quality, each stand‐alone analysis may not fully explain. In particular, the contribution of energy metabolism to sensory traits such as sweetness has not been fully elucidated. Since postmortem muscle metabolism is known to affect flavor development, investigating how dietary interventions influence energy‐related pathways may provide key insights into flavor enhancement mechanisms. Therefore, we employed a comprehensive metabolomics approach using Liquid Chromatography‐Mass Spectrometry (LC–MS) to analyze the taste‐related compounds and metabolites in beef. In recent years, metabolomics has been applied in foods (Muguruma et al. 2022; Abbiss et al. 2020), and several applications of metabolomics in beef have been reported (Chen et al. 2024; Muroya et al. 2020).

In this study, we combined a sensory evaluation with chemical analytical methods such as taste sensor analysis (Wu et al. 2020) and a comprehensive metabolomic analysis using LC–MS to investigate flavor differences between SL‐fed and control cattle.

## 2 Materials and Methods

The cattle used in the study were Japanese black cattle only A5‐ranked fattened on seven farms in Fukushima Prefecture. In the SL feeding group (SL), powdered sake meal produced by a local brewery was added to feed for 90 days (100 g/head/day) to cattle. In the control group, the cattle raised normally by the same farmers were used. After slaughtering the sample cattle, the longissimus thoracis muscles containing intramuscular fat were collected from the section located between the 6th and 7th ribs, extending 30 to 40 cm toward the caudal side, which is a typical ribeye portion used for meat quality evaluation. These samples were stored refrigerated at 4°C for about 14 days (14 or 15 days) after slaughter for analytical sensory evaluation, taste sensor analysis, and UPLC–MS/MS. Also, for glycogen quantitation, the longissimus thoracis muscle samples were kept refrigerated at 4°C for 16 days after slaughter.

### 2.1 Analytical Sensory Test

Analytical sensory test was performed by The Japan Food Analysis Center (JFAC, Japan) on behalf. The 12 trained panelists evaluated differences and characteristics of samples. We compared 4 samples from SL and 4 samples from control. All of these samples were collected from different individuals. Samples were formed into small pieces measuring 4 × 1 cm and transported frozen to the JFAC. After thawing in a refrigerator at 4°C from the day before the evaluation date, they were cooked in a convection oven at 200°C for 4 min. The panelists evaluated eight attributes: tenderness, juiciness, mouthfeel, *umami*, sweetness, fat sweetness, richness, and fat residuality, using a 7‐point scale (−3 to +3) with the control group set as 0. The scores of each panelist were summed for each evaluation item. Therefore, the maximum possible score for each item was 36 points (3 × 12 panelists).

### 2.2 Taste Sensor Analysis

In taste sensor analysis, a taste sensor modeled after the human tongue was used to quantify basic tastes: *umami*, *umami* richness, bitterness, bitterness and off‐flavor, astringent stimulation, salty, sour, sweet, and astringent. The cattle were used as independent samples: SL group (*n* = 20) and control group (*n* = 21). All of these samples were collected from different individuals. Beef samples were cut into 1 × 1 cm shapes, and 100 g were measured into a beaker. Beef samples were diluted 2.5 times with purified water and boiled in hot water for 60 min. After the hot water bath, the sample was cooled in ice water for 30 min, filtered through gauze into a 45 mL centrifuge tube, centrifuged at 845 *g* for 10 min, and the supernatant was used as the sample for measurement. The analysis was performed using TS‐5000Z (Intelligent Sensor Technology Corporation, Kanagawa, Japan).

### 2.3 Quantitation of Glycogen

Quantitation of glycogen was analyzed by EnzyChrom Glycogen Assay Kit (BioAssay Systems, USA), which is based on enzymatic hydrolysis of glycogen to glucose, followed by a colorimetric detection of the generated glucose at 570 nm. The cattle were used as independent samples: SL group (*n* = 20) and control group (*n* = 18). All of these samples were collected from different individuals. About 1 g was taken from the center of the beef sample and minced in a mortar. A 25 mg portion of the minced sample was placed into a finger masher and homogenized in 25 mM citrate buffer (pH 4.2), 2.5 g/L NaF on ice. Centrifuge at 14,000 *g* for 5 min to remove debris and use 10 μL clear supernatant for the assay. Assay operations were performed according to the kit protocol. Detection was performed by the colorimetric method.

### 2.4 Preparation of Beef Samples to UPLC–MS/MS

The cattle were used independent samples: SL group (*n* = 20) and control group (*n* = 18). All of these samples were collected from different individuals. Approximately 1 g of beef was collected from the center of the sample and ground using a mortar. A 50 mg aliquot of the homogenized sample was transferred to a 5 mL tube, mixed with 5 mL of 70% methanol, homogenized, and then sonicated for 5 min. The mixture was centrifuged, and 1 mL of the supernatant was transferred to a 2 mL tube. An equal volume (1 mL) of chloroform was added, and the mixture was vortexed for 30 s.

After centrifugation (4600 *g*, 5 min, 10°C), 400 μL of the upper aqueous phase was collected. This extract was filtered using an Amicon Ultra 5 K centrifugal filter (Merck Millipore, US) (9100 *g*, 120 min, 10°C), and the resulting filtrate was transferred to an LC–MS vial for analysis.

Metabolomic profiling was performed using a Nexera UHPLC system (Shimadzu, Kyoto, Japan) equipped with a Discovery HS F5 HPLC column (5 cm × 2.1 mm, 3 μm; Merck), coupled to an Impact II Q‐TOF mass spectrometer (Bruker Daltonics, Germany). The mobile phases consisted of 0.1% formic acid in water (A) and 0.1% formic acid in acetonitrile (B), with a flow rate of 0.25 mL/min. The gradient was programmed as follows: 0% B for 2 min; ramp to 65% B over 5 min; increase to 95% B by 15 min and hold until 20 min.

Solutes were detected using an ImpactII Q‐TOF‐MS/MS (Bruker, Germany). Electrospray ionization (ESI) was employed in both positive and negative ionization modes. The instrument was operated under the following conditions: Capillary voltage: 4500 V, Nebulizer gas (N^2^): 2.0 bar, Dry gas flow: 10.0 L/min, Dry gas temperature: 250°C, Collision energy: 10 to 50 eV (for MS/MS analysis), Scan range: m/z 50 to 1500, Lock mass calibration was achieved using sodium formate. A total of 54 standard compounds, including amino acids, nucleotides, and organic acids, were purchased from GL Sciences (Tokyo, Japan) as part of a commercial metabolite mix. Retention times and MS/MS spectra of these standards were used to construct an in‐house reference database (Data S1). Identification of metabolites was based on matching m/z values (within ±2.0 ppm) and retention times (±0.1 min) against this library. Additional validation was performed using commercial databases, including MetaboBASE Personal Library 3.0, HMDB Metabolite Library 2.0, and MoNA‐GNPS, with matching tolerances of m/z (2.0 ppm), mSigma (< 20), and MS/MS score (> 900). Relative quantification was performed using L‐methionine sulfone (Sigma‐Aldrich, USA) as an internal standard. Peak intensities were normalized to the internal standard, and results were expressed as relative abundance (arbitrary units). In targeted analysis, compounds were verified using authentic standards. In non‐targeted analysis, identification relied on accurate mass, isotope pattern, and MS/MS spectra.

### 2.5 Statistical Analysis

Metabolomic analysis of beef samples from SL‐fed and control cattle was conducted using LC–MS/MS. Both targeted and non‐targeted approaches were applied. Metabolite intensities were normalized using L‐methionine sulfone (Sigma‐Aldrich, USA) as an internal standard.

In the targeted analysis, metabolites were identified based on exact mass and isotope pattern matching and validated using retention times compared with those of authentic standards. In the non‐targeted analysis, metabolite identification was based on exact mass, isotope pattern, and MS/MS fragmentation, and confirmed by comparison with commercial databases including MetaboBASE Personal Library 3.0, HMDB 2.0, and MoNA‐GNPS.

To evaluate global differences in metabolic profiles between SL‐fed and control groups, partial least squares discriminant analysis (PLS‐DA) was performed. Metabolites with high factor loadings were considered to be key contributors to group separation. Volcano plot analysis was used to identify metabolites that showed statistically significant differences between the two groups, based on fold change and *p*‐values. For individual metabolite comparisons, unpaired *t*‐tests were performed. Pathway enrichment analysis was conducted to determine whether differentially abundant metabolites were overrepresented in specific metabolic pathways.

## 3 Results and Discussion

The results of the analytical sensory test based on human perception comparing the SL group with the control are shown in Table 1. The evaluation method was conducted by setting the control group's evaluation score to 0 and scoring the SL on a 7‐point scale (−3 to +3). The SL group showed significantly higher scores than the control in *Juiciness*, *Tenderness* (softness), *Sweetness*, and *Fat sweetness*, with *Juiciness* receiving the highest score (19 points) among all evaluated attributes. In contrast, *Umami*, Mouthfeel, Richness, and *Fat residuality* did not show significant differences between the groups, although all tended to be higher in the SL group. These findings suggest that SL feeding improves specific aspects of beef palatability—particularly those related to softness, sweetness, and juiciness—as perceived by human sensory evaluation.

**TABLE 1:** The results of the analytical sensory test score of the SL group (when control is set to 0). *p* < 0.05 with unpaired *t*‐test.

	Tenderness (softness)	Juiciness	Mouthfeel	Umami	Sweetness	Fat sweetness	Richness	Fat residuality
Score	16	19	12	8	13	17	9	6

The taste sensor analysis, which provides an objective mechanical evaluation, detected *umami*, *umami richness*, and *sweetness*. As shown in Table 2, *the sweetness* value in the SL group reached 0.5 relative to the control (set to 0), a level typically perceptible to individuals with a keen sense of taste. However, this difference was not statistically significant (*p* = 0.089). While *umami* (*p* = 0.255) and *umami richness* (*p* = 0.626) values remained below perceptible thresholds, the observed trend toward increased *sweetness* is consistent with the results of the analytical sensory test, supporting the hypothesis that SL feeding may enhance the sweetness of beef. Since the sample used for taste sensor analysis is a water‐soluble extract, the sweet taste of beef is mainly derived from water‐soluble compounds. Sugars, sugar alcohols, amino acids, and peptides are representative water‐soluble compounds that impart sweetness. Among these, sugars are well‐established contributors to *sweetness* in beef.

**TABLE 2:** Analytical sensory testing value of SL group (when control is set to 0).

Umami richness	Umami	Sweetness
0.1	0.2	0.5

**FIGURE 1:**
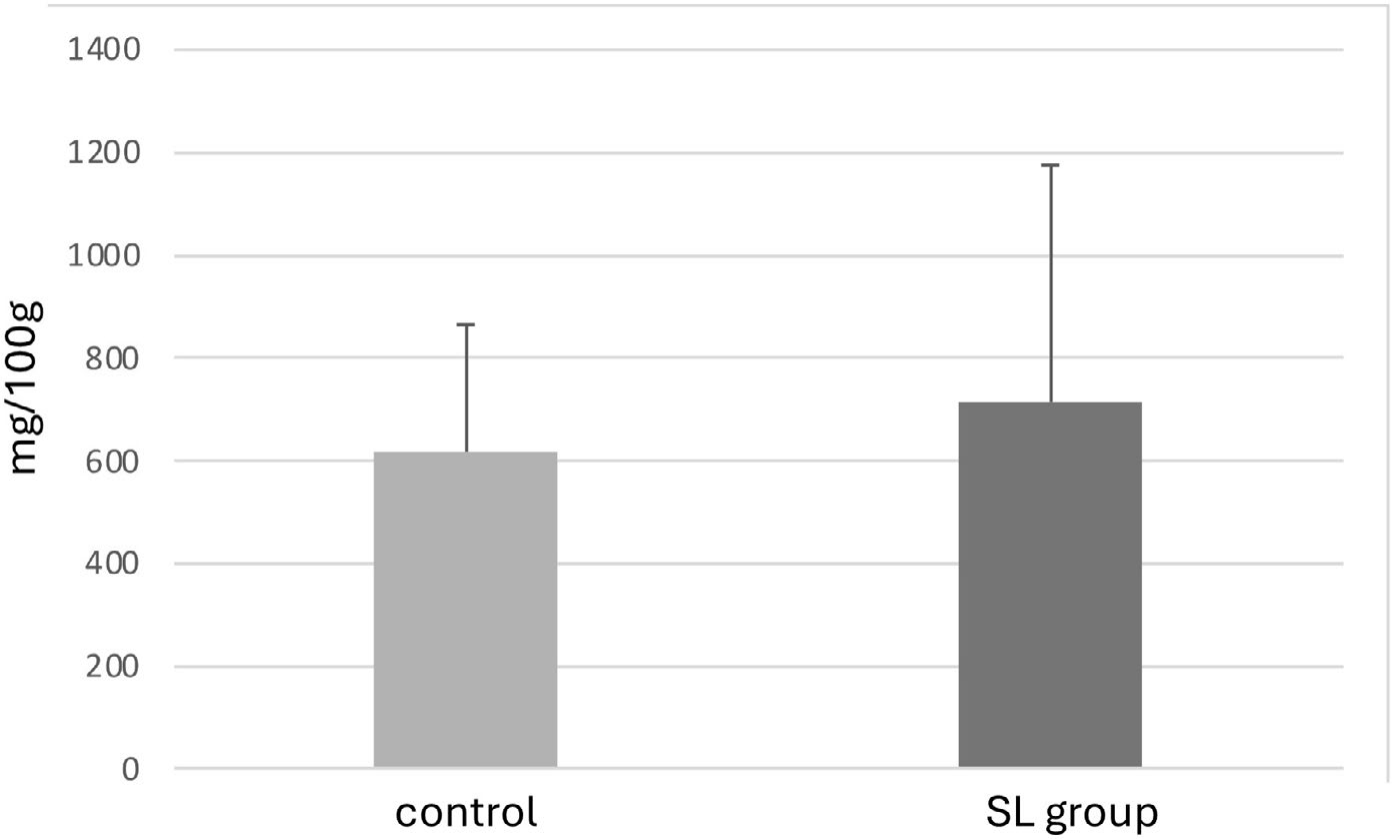
Glycogen content in 100 g of beef with SL group and control. The cattle were used independent samples: SL group (*n* = 20) and control group (*n* = 18).

Therefore, to explore whether sugar‐related compounds might be responsible for the observed sweetness, we focused on glycogen, a major carbohydrate reserve in muscle that can be enzymatically converted into glucose. Although not statistically significant (*p* = 0.46), the observed difference suggests a possible trend toward an increase in glycogen level in the SL group. However, further validation with a larger sample size is warranted (Figure 1). In this analysis, glycogen was hydrolyzed into glucose by enzymatic reaction and quantified as glucose equivalents. Previous studies have reported that glycogen decreases in muscle after slaughter, while glucose—its metabolite—increases during postmortem aging (Rosenvold et al. 2001; Komatsu et al. 2020). These findings suggest that the enhanced sweetness observed in the SL group may, at least in part, reflect greater glycogen availability at the time of slaughter, contributing to elevated glucose levels postmortem. This observation led us to further investigate whether energy‐related metabolic pathways might be differentially regulated in SL‐fed beef.

### 3.1 LC–MS/MS Analysis of Beef Extract and Statistical Analysis

**FIGURE 2:**
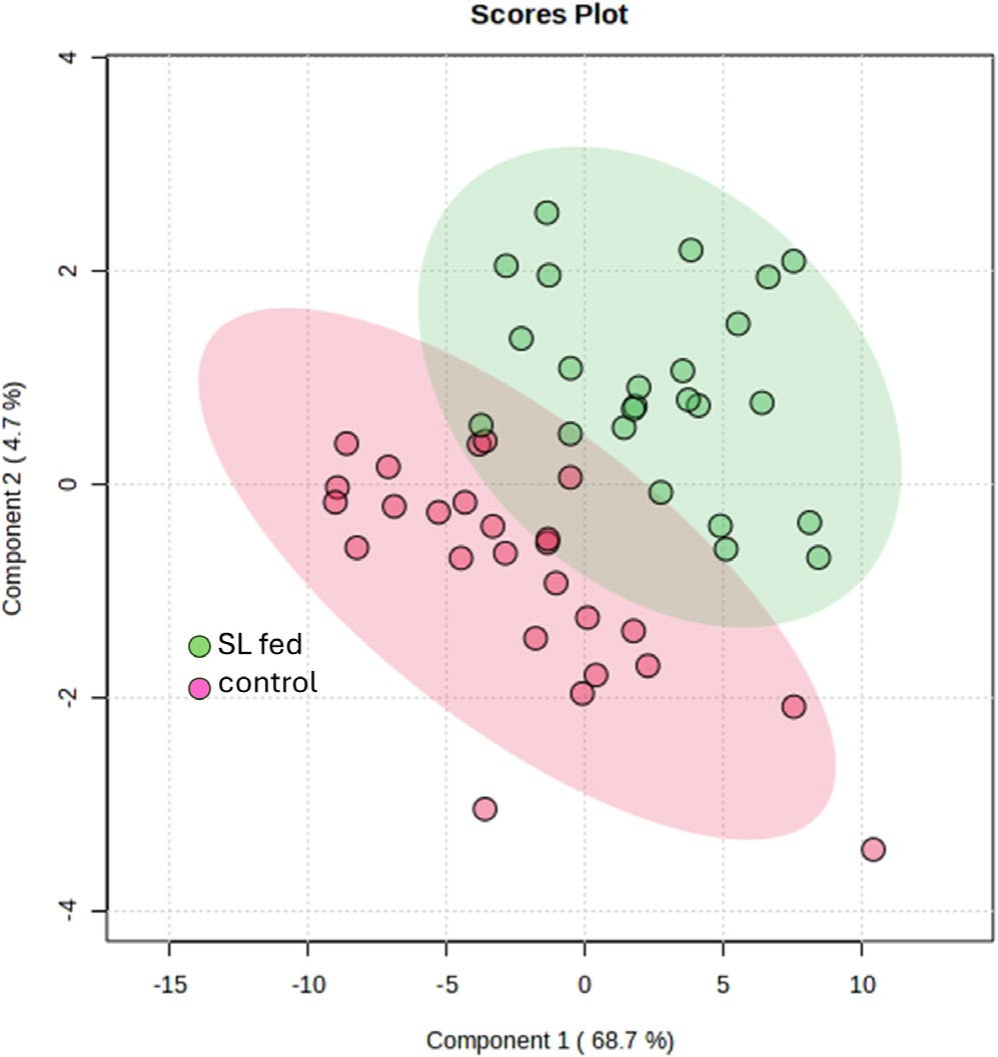
Beef extraction metabolite levels of SL group and control. PLS‐DA score plot showing that beef of SL group and control formed different clusters. The cattle were used independent samples: SL group (*n* = 20) and control group (*n* = 18).

LC–MS/MS results of the beef extract identified 52 compounds, based on reference standards of 54 compounds and complementary database matching. We conducted a statistical analysis of beef extract metabolite profiles to identify compounds related to flavor differences between SL‐fed and control groups. Partial least‐squares discriminant analysis (PLS‐DA), which included the levels of 39 metabolites (see Figure 5) obtained from targeted and non‐targeted metabolome analysis as variables, revealed that beef from the SL group and the control formed distinct clusters, indicating that a well‐defined separation exists between the groups—particularly along the PLS1 axis—and suggesting that SL feeding introduces a systematic shift in the metabolic profile (Figure 2). Notably, the control group displays broader dispersion, indicating natural variability in the absence of intervention, while the SL group shows a tendency to cluster more centrally, implying a convergence toward a consistent metabolic state. This suggests that SL feeding not only alters the metabolome but may also reduce variability in beef quality across individual cattle.

**FIGURE 3:**
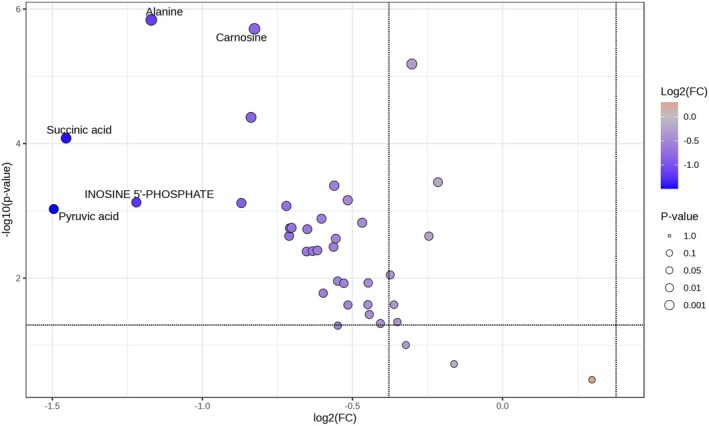
Volcano plot showing significantly upregulated metabolites in beef from SL‐fed cattle compared to control. Metabolites located in the upper left quadrant (*p* < 0.05, log_2_ fold change < −0.4) are considered significantly increased in the SL‐fed group.

Volcano plot analysis showed significant differences in 29 compounds (Data S2), with alanine, identified in the targeted analysis, being the most significantly different metabolite in the SL group and control (Figure 3). In this Volcano plot, the x‐axis represents log_2_(Control/SL), such that metabolites significantly increased in the SL group appear on the left side of the plot. Alanine, a sweet‐tasting amino acid, was the most upregulated compound.

The Volcano plot also revealed significant upregulation of succinic acid, pyruvic acid, carnosine, and inosine 5′‐phosphate (IMP) in the SL group. Succinic acid is known to impart mild *umami* and salty flavors and contributes to flavor persistence, while pyruvic acid plays a role in sourness (Li and Liu 2015) and serves as a precursor to aroma‐related compounds, potentially influencing the flavor complexity of cooked beef. Carnosine, a dipeptide abundant in muscle, is classically associated with sweetness and enhances flavor depth and reduces bitterness, contributing to overall palatability (Gauri et al. 2024). IMP is known as an *umami*‐related compound.

**FIGURE 4:**
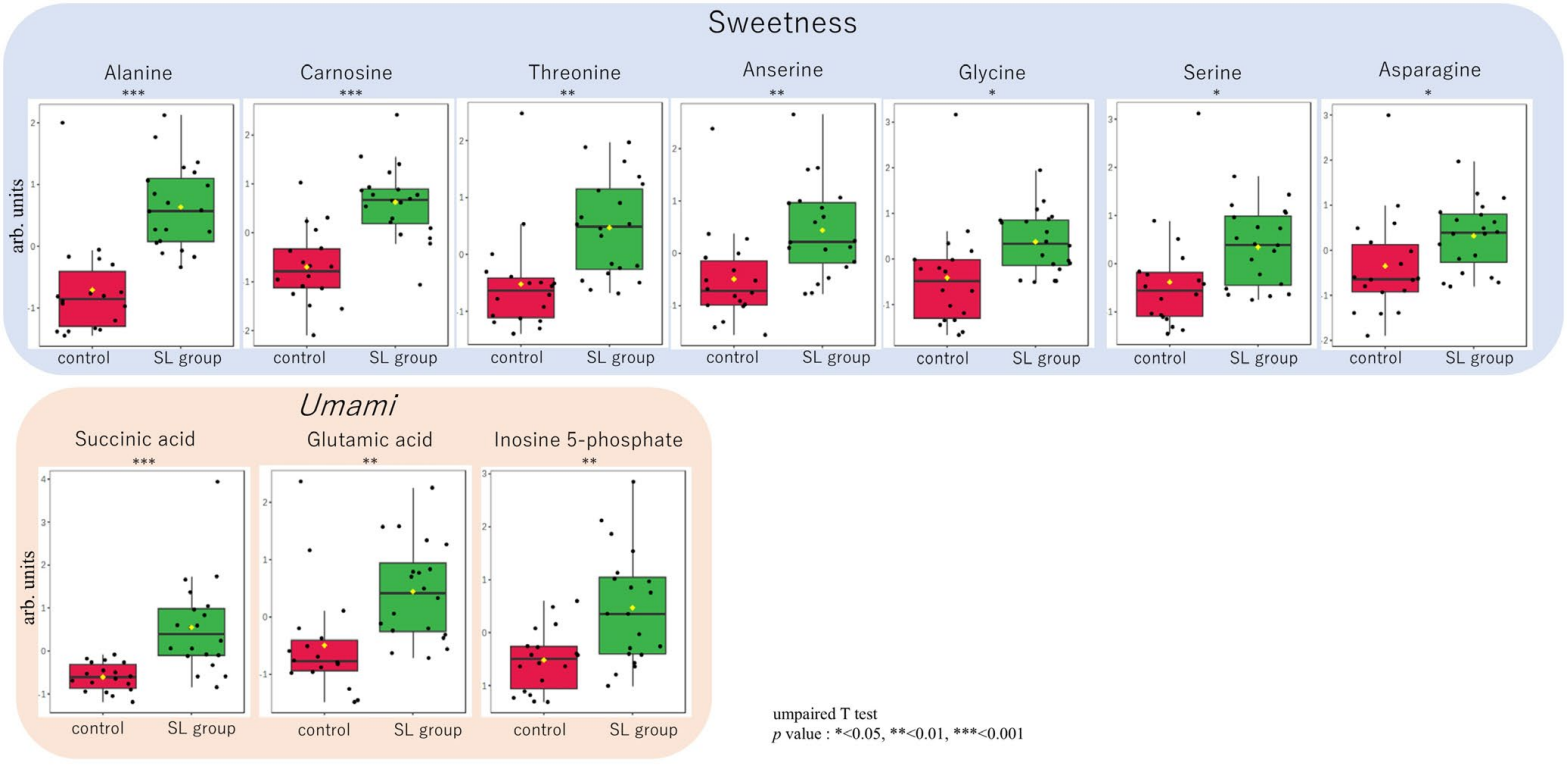
Metabolites of sweetness and *umami* showed statistical differences in levels between SL group and control. Box of red; control, green; SL group.

Among the compounds significantly increased in the Volcano plot, sweet‐ and *umami*‐tasting compounds (You et al. 2024; Suyama and Shimizu 1982) are shown in the boxplots in Figure 4. There were seven sweetness‐related compounds (Alanine, Carnosine, Threonine, Anserine, Glycine, Serine, Asparagine), all of which were amino acids or oligopeptides. *Umami*‐related compounds included succinic acid, glutamic acid, and IMP. Taste sensor analysis also detected sweetness, *umami*, and *umami* richness. While no significant differences were observed in *umami* and *umami* richness between groups, glutamic acid and IMP are known to exhibit a synergistic effect, in which their combination enhances *umami* perception to a level more than eight times stronger than either compound alone (Kurihara 2015). This suggests that a greater difference in *umami* perception could emerge upon actual consumption, even in the absence of statistical significance in sensor data.

**FIGURE 5:**
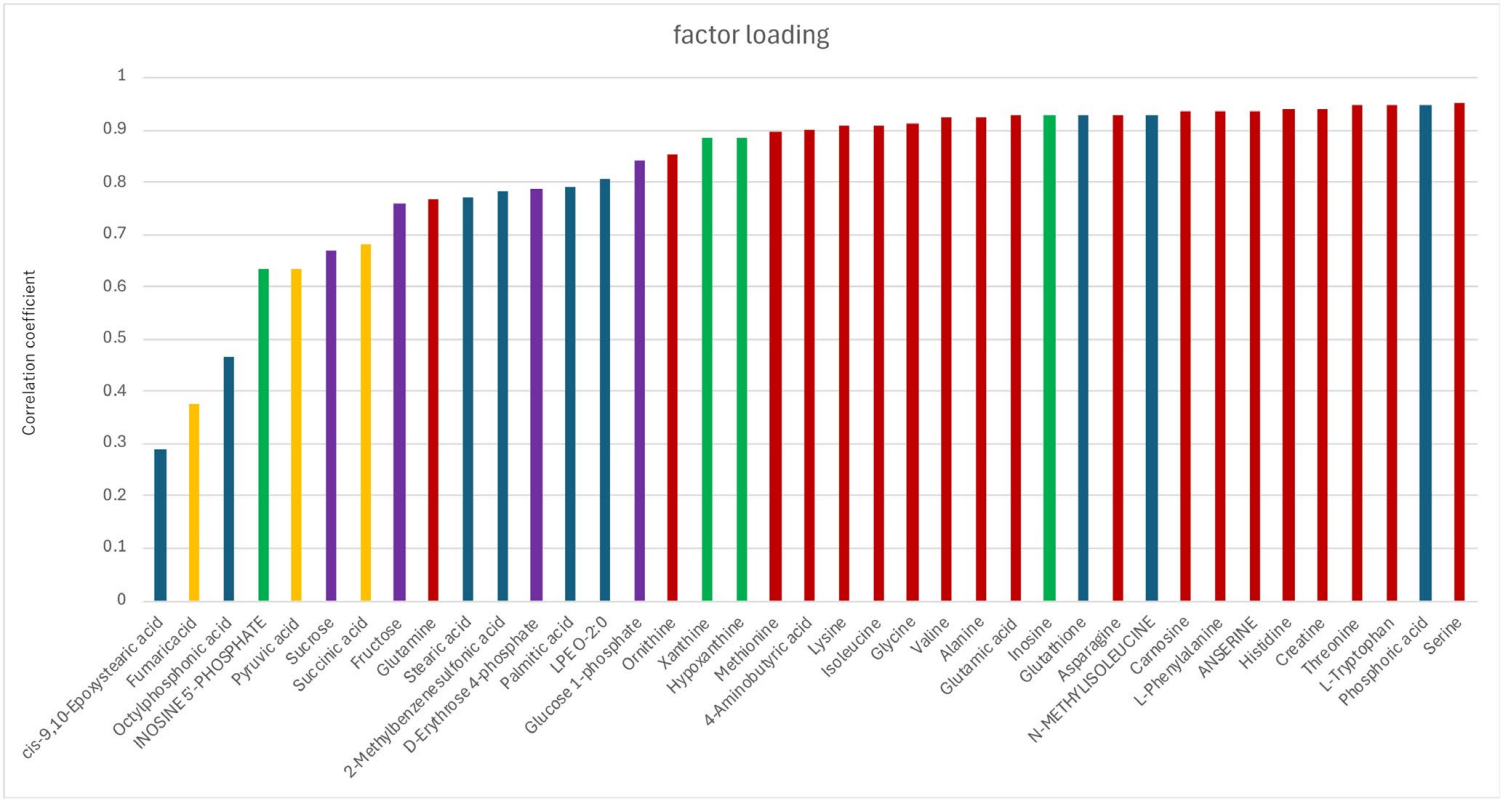
Factor loadings calculated from the VIP score of the PLS‐DA. X‐axis is the correlation coefficient for compounds that are increased in SL group. Bar of red; amino acids and oligo‐peptides, green; nucleotides, purple; sugar related compounds, yellow; organic acids, blue; other metabolites.

The factor loadings in the PLS‐DA are shown in Figure 5. Among the 39 compounds included in the analysis, 19 were amino acids (red bar), 4 were nucleotides (green bar), 4 were sugar‐related compounds (purple bar), 3 were organic acids (yellow bar), and 9 belonged to other metabolites (blue bar). In general, a variable is considered particularly important when the factor loading exceeds 0.7 and strongly contributing when it exceeds 0.5. Serine showed the highest factor loading among all compounds, and several other amino acids also had values greater than 0.7. Furthermore, three nucleotides showed loading above 0.8, indicating that they were major contributors to the group separation. Since amino acids and nucleotides are known to play a major role in taste perception, their strong loadings suggest that these compounds characterize the SL group's distinct taste profile.

Among the sugar‐related compounds, three showed loadings above 0.7, implying their involvement in sweetness. Two organic acids had loadings greater than 0.5 and are thought to contribute to sourness, bitterness, or saltiness. Of the nine compounds classified as “others” seven exhibited high factor loadings above 0.7, suggesting their potential importance in group discrimination.

**FIGURE 6:**
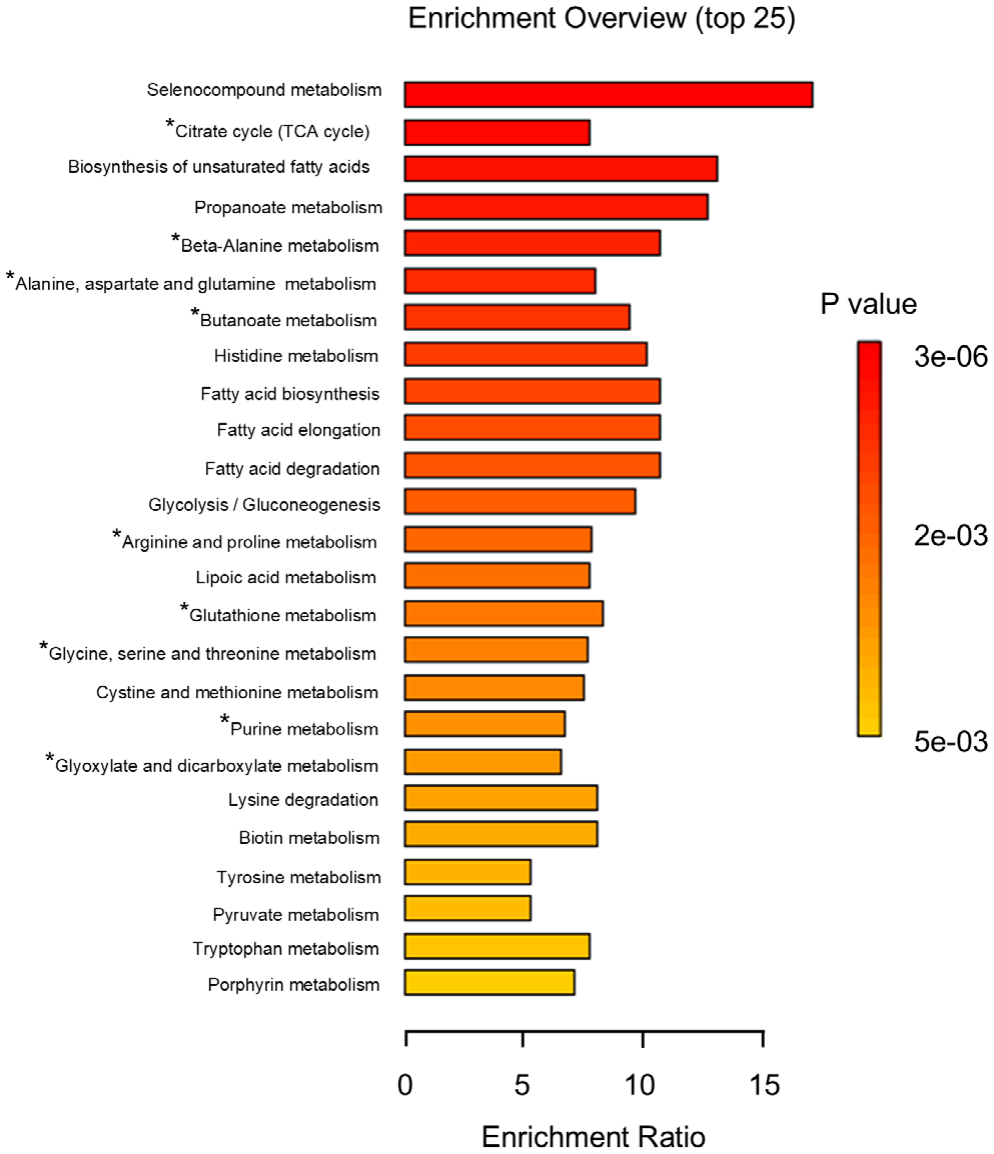
Enrichment analysis was performed with the KEGG database in beef with SL group and control. *; Metabolic pathways in which 3 or more metabolites.

Enrichment analysis was performed to identify metabolic pathways significantly affected by SL feeding, based on enrichment ratio and *p*‐value. Among the top 25 enriched pathways (Figure 6), there is an enrichment of multiple energy‐related pathways, including the TCA cycle and amino acid metabolism. We hypothesized that SL feeding may enhance energy availability in muscle tissue, potentially influencing glycogen metabolism.

**FIGURE 7:**
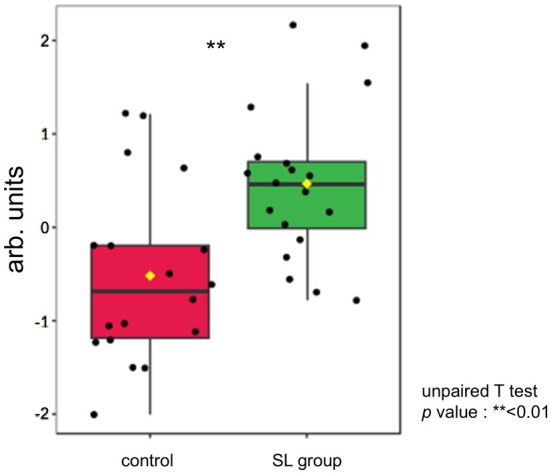
G1P showed statistical difference in level between SL group and control. The cattle were used independent samples: SL group (*n* = 20) and control group (*n* = 18).

Among the enriched energy‐related pathways, the citrate cycle (TCA cycle) showed the highest enrichment, followed by Beta‐Alanine metabolism, Alanine, Aspartate and Glutamate metabolism, Butanoate metabolism, Glycine, Serine and Threonine metabolism, and Glyoxylate and Dicarboxylate metabolism. All these pathways were more active in the SL group, supporting the idea that energy production was enhanced. In particular, Butanoate metabolism and glyoxylate and dicarboxylate metabolism are considered to be involved in ruminal fermentation (Zhuang et al. 2024; Guo et al. 2019), suggesting that metabolic changes may begin in the rumen and extend to systemic energy metabolism. Enhanced energy production in the SL group is expected to promote glycogen synthesis. Although there were no statistically significant differences, glycogen quantification results tended to be higher in the SL group. Glucose 1‐phosphate (G1P) showed a significant difference; which is a key intermediate in glycogen metabolism, is produced during glycogen breakdown and is essential for glycogen resynthesis (Figure 7). Glycogen is primarily stored in the liver and muscle, but postmortem, lactic acid is produced by obtaining G1P. This process continues for approximately 24 to 48 h after slaughter, during which some glycogen remains. According to Immonen et al. (Immonen and Puolanne 2000), approximately 45% of the original glycogen remains in beef muscle 4 days after slaughter. During this postmortem period, G1P also persists in muscle tissue and may reflect the metabolic state at slaughter. We hypothesize that the activation of energy‐producing pathways, such as the TCA cycle, leads to increased ATP generation—which in turn promotes energy storage—SL feeding may have facilitated glycogen resynthesis through increased metabolic activity.

These findings suggest that enhanced energy metabolism induced by SL feeding contributes to glycogen retention or synthesis in muscle tissue. Additionally, purine metabolism was significantly higher in the SL group. Five purine metabolites were detected, among which inosine 5′‐monophosphate (IMP) is particularly notable for contributing to *umami* taste, consistent with the analytical sensory test that showed a trend toward higher *umami* in the SL group.

## 4 Conclusion

These results suggest that feeding SL to black Japanese beef can alter the taste of the beef in a way that is perceptible to humans, particularly enhancing sweetness, softness, and juiciness. The key contributing compounds identified in this study were amino acids, oligopeptides, and nucleotides, all of which were found to play an important role in these taste improvements.

Comprehensive metabolomics analysis, which considered changes in multiple metabolic pathways, indicated that the consumption of SL stimulates energy production, potentially promoting glycogen synthesis. By combining taste evaluation through both analytical sensory tests and taste sensor analysis with in‐depth metabolomic insights, this study provides scientific evidence of the mechanisms behind taste changes.

This approach is highly useful not only in the development of livestock fattening technologies but also in the broader field of food science and quality enhancement.

## Author Contributions

**Hitomi Shikano and Shu Taira:** conceptualization (lead), formal analysis (lead), investigation (lead), methodology (lead), writing – original draft (equal). **Kazuki Komatsu:** data curation (supporting). **Fumiya Koga:** data curation (supporting). **Meguru Hara:** investigation (supporting). **Kazuaki Yoshinaga:** formal analysis (equal), investigation (equal). **Naoto Ishikawa:** project administration (lead).

## Conflicts of Interest

The authors declare no conflicts of interest.

## Supporting information


**Data S1:** Supporting Information.

**Data S2:** Supporting Information.

## Data Availability

The data that support the findings of this study are available within the article and its Supporting Information.
