# Supplementary figures and images for: Taste Enhancement in Japanese Black Wagyu Beef Fed With Sake Lees: Insights From Metabolomic and Sensory Evaluations
Source: Food Sci Nutr. 2025 Aug 25;13(9):e70839. doi: 10.1002/fsn3.70839 (PMC12378072; doi:10.1002/fsn3.70839)

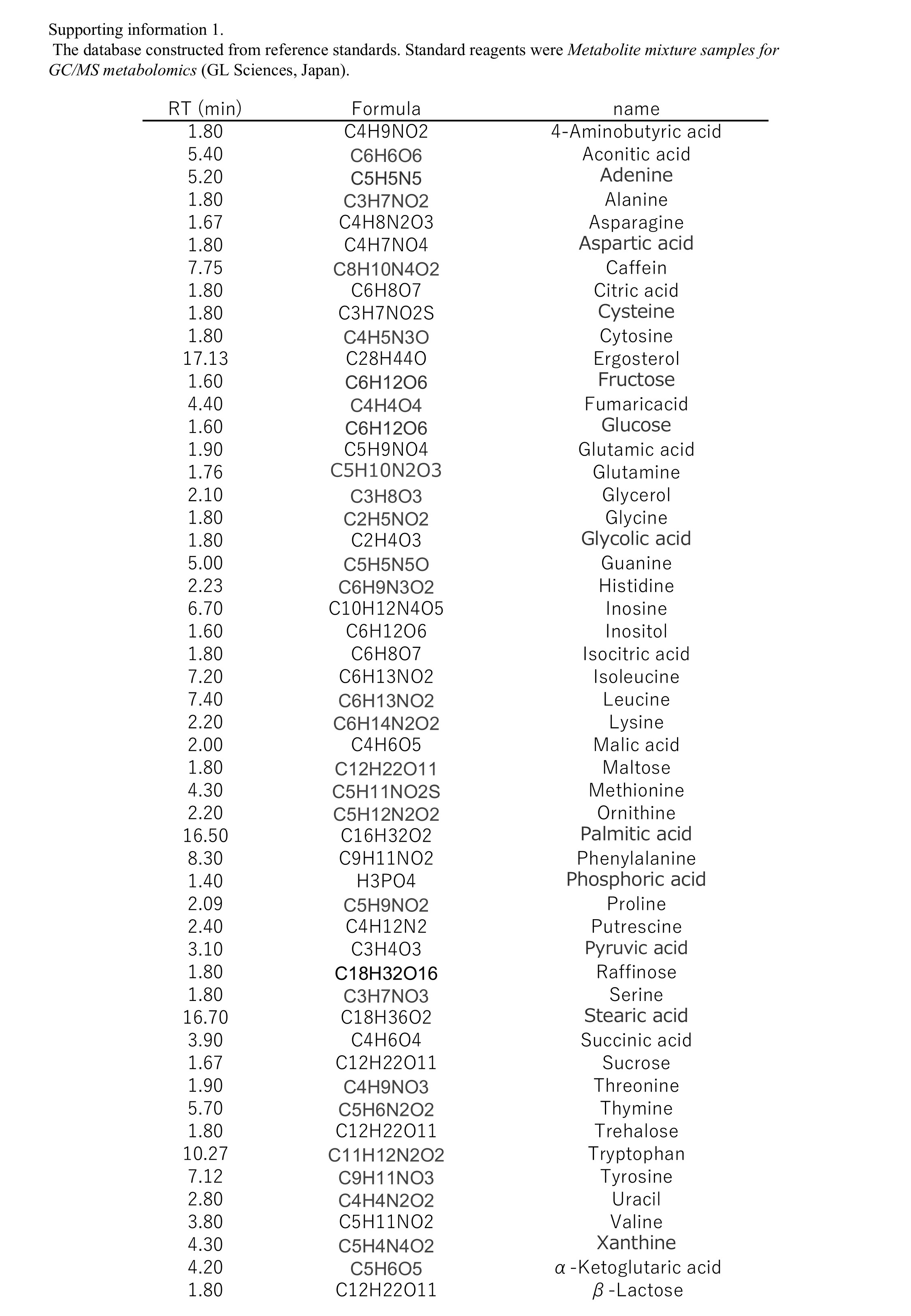

Supplement: Supplementary file 1 — Data S1: Supporting Information. [file FSN3-13-e70839-s002.jpg]

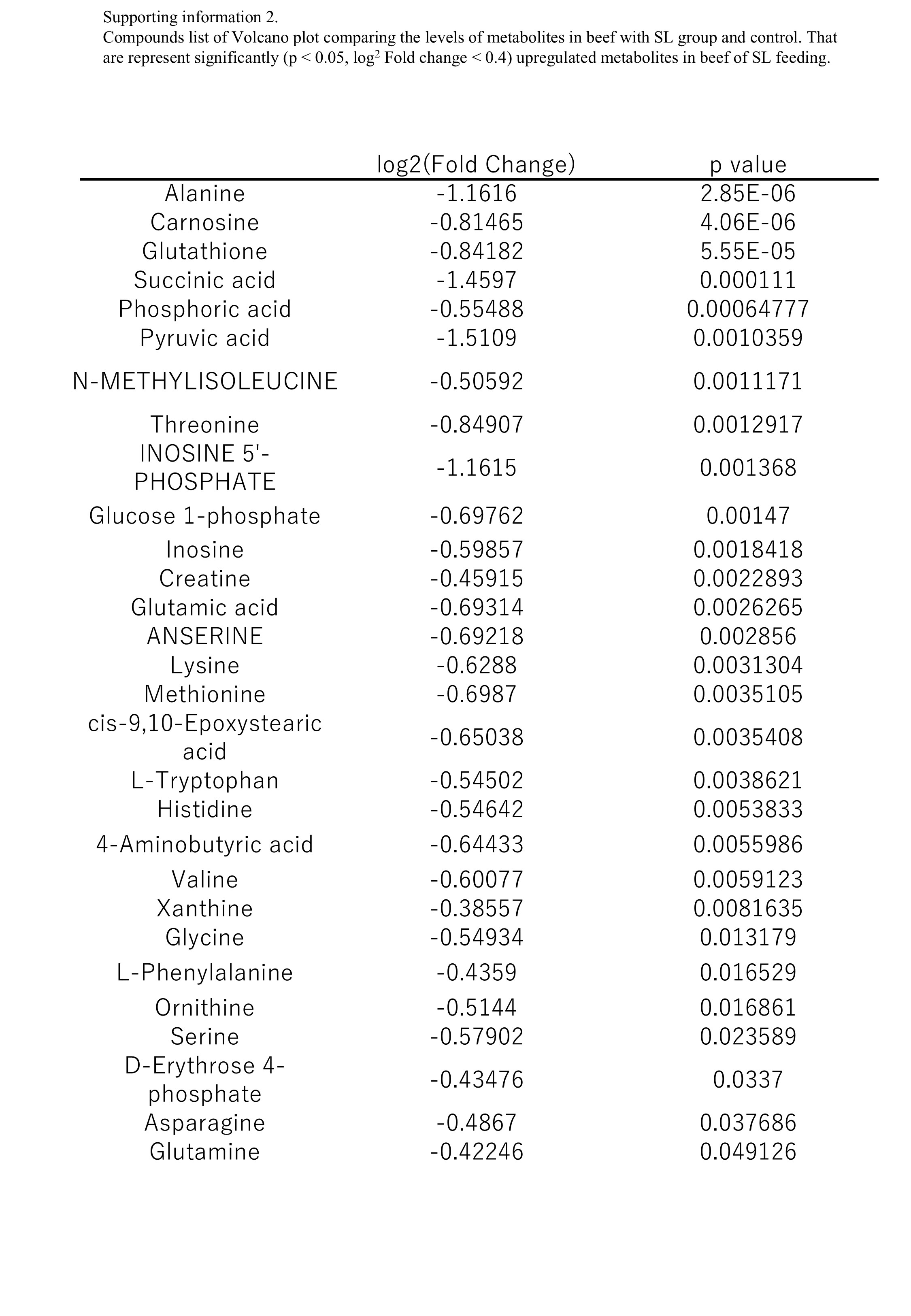

Supplement: Supplementary file 2 — Data S2: Supporting Information. [file FSN3-13-e70839-s001.jpg]
